# Handling misclassified stratification variables in the analysis of randomised trials with continuous outcomes

**DOI:** 10.1002/sim.9818

**Published:** 2023-06-26

**Authors:** Lisa N. Yelland, Jennie Louise, Brennan C. Kahan, Tim P. Morris, Katherine J. Lee, Thomas R. Sullivan

**Affiliations:** 1 Women and Kids Theme, South Australian Health and Medical Research Institute, Adelaide, South Australia, Australia; 2 School of Public Health, The University of Adelaide, Adelaide, South Australia, Australia; 3 Adelaide Medical School, The University of Adelaide, Adelaide, South Australia, Australia; 4 MRC Clinical Trials Unit at UCL, London, UK; 5 Clinical Epidemiology and Biostatistics Unit, Murdoch Children’s Research Institute, Melbourne, Victoria, Australia; 6 Department of Paediatrics, The University of Melbourne, Melbourne, Victoria, Australia

**Keywords:** covariate misclassification, covariate-adaptive randomisation, randomisation error, stratification error, stratified randomisation

## Abstract

Many trials use stratified randomisation, where participants are randomised within strata defined by one or more baseline covariates. While it is important to adjust for stratification variables in the analysis, the appropriate method of adjustment is unclear when stratification variables are affected by misclassification and hence some participants are randomised in the incorrect stratum. We conducted a simulation study to compare methods of adjusting for stratification variables affected by misclassification in the analysis of continuous outcomes when all or only some stratification errors are discovered, and when the treatment effect or treatment-by-covariate interaction effect is of interest. The data were analysed using linear regression with no adjustment, adjustment for the strata used to perform the randomisation (randomisation strata), adjustment for the strata if all errors are corrected (true strata), and adjustment for the strata after some errors are discovered and corrected (updated strata). The unadjusted model performed poorly in all settings. Adjusting for the true strata was optimal, while the relative performance of adjusting for the randomisation strata or the updated strata varied depending on the setting. As the true strata are unlikely to be known with certainty in practice, we recommend using the updated strata for adjustment and performing subgroup analyses, provided the discovery of errors is unlikely to depend on treatment group, as expected in blinded trials. Greater transparency is needed in the reporting of stratification errors and how they were addressed in the analysis.

## 1 Introduction

Stratified randomisation is a popular approach for randomly assigning participants to treatment groups in clinical trials.^1–3^ It is a member of a broader class of covariate-adaptive randomisation procedures^2^ that take baseline covariates into account when allocating participants to treatment groups, thus preventing imbalance between the randomised groups on the chosen covariates at the trial design stage.^4^ To implement stratified randomisation, participants are divided into subgroups or strata defined by one or more baseline covariates. Permuted block randomisation is then performed within each stratum, resulting in treatment groups that are closely balanced with respect to the stratification variables. Stratified randomisation has many advantages compared to simple randomisation, particularly in small trials, including improved precision of the treatment effect estimate, and enhanced credibility of the trial.^4,5^

It is widely recognised that analytic methods should reflect the trial design and hence adjustment should be made for the stratification variables in the analysis of trials that use stratified randomisation.^5–9^ Kahan and Morris^10^ provided an intuitive rationale for performing this adjustment by showing that stratification induces correlation between the sample means of a continuous outcome in the intervention and control groups. Failing to account for this correlation by performing an unadjusted analysis leads to a range of problems when estimating treatment effects, including SEs that are too large, confidence intervals that are too wide, type I error rates that are too low and reduced power. In contrast, an adjusted analysis that includes the stratification variables as covariates in a regression model provides valid inference for treatment effects.^10,11^

When a stratification variable is affected by measurement error, some participants may be misclassified and hence randomised in the incorrect stratum. Such errors, referred to hereafter as *stratification errors*, can occur for many reasons. For instance, participants may incorrectly report the value of their stratification variable, or researchers may incorrectly enter the value into a web-based randomisation service.^12^ While stratification errors are rarely reported in trial publications,^3^ error rates as high as 17% have been documented.^13^ These errors raise the question of whether the analysis should be adjusted for the strata used to perform the randomisation (the *randomisation strata*), which may be incorrect for some participants but ensures the analysis reflects the trial design, or the strata defined by the true covariate values (the *true strata*), which are expected to have a stronger relationship with the outcome. Adjusting for the true strata is only possible if all stratification errors are identified and corrected prior to analysis, though one can rarely be certain this is the case. In practice, some stratification errors may go unrecognised and hence a choice must be made between adjusting for the randomisation strata or the strata that have been updated to correct errors discovered prior to analysis (the *updated strata*).

Several previous studies have explored the impact of stratification errors on treatment effect estimation using regression models. Ke et al.^13^ studied continuous and time-to-event outcomes. Their simulation study identified no bias or power reduction when adjusting for the true strata but found that error variances were overestimated, power was reduced, and treatment-by-covariate interaction effect estimates were biased when adjusting for the randomisation strata. They suggested adjusting for the randomisation strata in the analysis, adjusting for the true strata in a sensitivity analysis when the error rate is large, and using the true strata to perform subgroup analyses by the stratification variable. Fan et al.^14^ studied binary and count outcomes. Their simulation study also showed that adjusting for the true strata was optimal, while adjusting for the randomisation strata resulted in biased conditional treatment effect estimates (for binary outcomes only) and reduced power. They recommended adjusting for the true strata in the analysis. Most recently, Wang et al.^15^ showed theoretically that adjusting for the randomisation strata achieves the nominal type I error rate for continuous outcomes. They did not consider adjusting for the true strata or provide any recommendations to guide practice. Importantly, none of these previous studies considered the performance of different adjustment approaches in the realistic setting where stratification errors occur but only some errors are discovered. This limitation, together with the lack of clear and consistent recommendations for practice, may leave applied researchers unsure how best to analyse data from clinical trials affected by stratification errors.

The aims of the current article are: (1) to explore the impact of stratification errors on the correlation between sample means induced by stratification; and (2) to compare methods of adjusting for stratification variables affected by misclassification in the analysis of continuous outcomes, when all or only some stratification errors are discovered, and when the treatment effect or treatment-by-covariate interaction effect is of interest. In Sections 2 and 3, we describe the simulation methods and results that address our aims. In Section 4, we present an example trial where around 4% of participants were randomised in the incorrect stratum to illustrate the impact of choosing different analysis methods on trial results in practice. We discuss our key findings in Section 5 and provide practical recommendations for researchers involved in the design and analysis of clinical trials that utilise stratified randomisation in Section 6.

## 2 Simulation Methods

A simulation study was conducted to investigate the impact of stratification errors on the analysis of trials that use stratified randomisation. Continuous outcomes for the *i*th patient were randomly generated from the model

(1) $$Y_{i}\;=\;\alpha \;+\;\beta_{T}T_{i}\;+\;\beta_{X}X_{i}\;+\;\beta_{TX}T_{i}X_{i}\;+\;e_{i},$$

where *T_i_* is a binary indicator for the randomised treatment group (*T_i_* = 0 for patients assigned to the control arm and *T_i_* = 1 for patients assigned to the intervention arm), *X_i_* is a binary baseline covariate with prevalence 0.5 that was randomly generated from a Bernoulli (0.5) distribution, and *e_i_*~*N* (0, 1) is a residual error term. The sample size was fixed at 1000 based on power considerations (see below) and treatment group was assigned using randomly permuted blocks of size 4 (1:1 randomisation) within strata defined by *Z_i_*, which differs from *X_i_* if the *i*th patient is affected by a stratification error and matches *X_i_* otherwise (ie, *Z_i_* is the randomisation stratum for the *i*th patient and *X_i_* is the true stratum). If a stratification error is discovered for the *i*th patient, the updated stratum is recorded in *W_i_*, which differs from *X_i_* if a stratification error occurs but remains undiscovered and matches *X_i_* otherwise.

For each scenario, 10 000 simulated datasets were generated and analysed using Stata 16.1 (StataCorp, College Station, Texas, USA). Simulation results were summarised using the simsum command^16^ and simulation code is provided in a Supplementary File S1. Further details for each specific simulation setting are provided below using the ADEMP framework for describing the Aims, Data-generating mechanisms, Estimands, Methods, and Performance measures for simulation studies.^17^ Simulation parameters (summarised in Table 1) were primarily chosen with the aim of identifying problems that may occur with particular adjustment approaches and hence may be more extreme than values typically encountered in real trials.

### 2.1 Effect of stratification errors on the correlation between treatment groups

The previous finding that stratification induces correlation between the sample mean outcomes in the treatment groups^10^ provides an intuitive explanation for why an unadjusted analysis that ignores this correlation performs poorly in the absence of stratification errors. It is, therefore, of interest to understand how stratification errors influence the correlation induced by stratification when interpreting the performance of an unadjusted analysis in the presence of stratification errors. To explore this issue, outcomes were generated from model (1) with *α* = *β_T_* = *β_TX_* = 0 (note that the correlation between treatment groups is independent of the treatment effect for a linear regression model^10^) and *β_X_* = 1 or 3 (ie, the mean outcome differs by 1 or 3 SDs between the two strata). The former value for *β_X_* was chosen as a large but realistic covariate effect (eg, many studies have reported mean differences of 1 SD or more in health outcomes of children born preterm vs those born at term^18–21^), while the latter value was chosen as an extreme covariate effect to produce a moderate correlation between the treatment group means in the absence of stratification errors^10^ and thus highlight the effect of stratification errors on this correlation. Stratification errors were introduced at random with probability ranging from 0 to 0.4 in increments of 0.05. The estimand of interest was the correlation between the mean outcome in each treatment group. To estimate this quantity, the sample mean was calculated by randomised treatment group within each simulated dataset and the correlation between the samplemeanswas then calculated across repetitions for each scenario (2 covariate effects × 9 stratification error probabilities = 18 scenarios). The Monte Carlo SE for the Fisher *z*-transformation of the correlation was calculated as $\sqrt{\text{1}/(\text{number}\;\text{of}\;\text{repetitions}\;-\;3)}\approx 0.01.$

### 2.2 Estimating treatment effects when all stratification errors are discovered

To compare the performance of different methods of adjusting for stratification variables when all stratification errors are discovered, outcomes were generated from model (1) with *α* = *β_TX_* = 0, *β_T_* = 0or 0.2, and *β_X_* = 1 or 3. The former value for *β_T_* was chosen to allow examination of the type I error rate, while the latter value was chosen to provide >80% power to detect a difference in means between the treatment groups for the fixed sample size of 1000 (power=88% based on a two-sample *t*-test with *α* = 0.05). The covariate effects *β_X_* were chosen to be relatively large, since stratification should ideally be based on the most prognostic covariates. In addition, there is little difference between an unadjusted analysis and an analysis adjusting for the stratification variable when the covariate effect is small^10^ and hence any differences in adjustment approaches would likely be difficult to see with smaller values of *β_X_*. Four stratification error patterns were considered: stratification errors occurring at random with probability 0.01 or 0.2 across both strata; and stratification errors occurring at random with probability 0.01 or 0.2 overall, such that errors were three times more likely in one stratum than the other (ie, the probability of a stratification error was 0.005 or 0.1 in stratum *X* = 0 and 0.015 or 0.3 in stratum *X* = 1). While a stratification error rate around 1% in all strata seems realistic for most trials, error rates approaching 20%^13^ or that vary across strata^14^ have been documented and should highlight any differences that may exist between adjustment methods for informing general recommendations. As stratification errors occur prior to treatment group allocation, errors were generated independent of treatment group for each of the 16 scenarios considered (2 treatment effects ×2covariate effects × 4 stratification error patterns). For reference, simulations were repeated with no stratification errors (2 treatment effects × 2 covariate effects = 4 scenarios).

Using *Y*(*t*)to denote the potential outcome when a participant is assigned treatment *t*, the estimand of interest for this simulation study was *E*[*Y*(1)-*Y*(0)],the expected difference in potential outcomes between the intervention and control arms, with true value *β_T_* as used in the data generation process. The data were analysed using a linear regression model with a main effect for the randomised treatment group and either:

1. no additional covariates (unadjusted analysis),
2. a main effect for *Z* (adjusted for the randomisation strata), or
3. a main effect for *X* (adjusted for the true strata, which equals the updated strata *W* in this setting where all stratification errors are discovered).

While adjusting for both *Z* and *X* within the samemodel is another possible analysis approach, thiswas not considered due to the high correlation between *Z* and *X*, particularly in the low error setting.

Letting $\hat{\beta_{Tj}}$ represent the treatment effect estimate for the *j*th simulated dataset (*j* = 1 … 10 000), results from each scenario were summarised across repetitions according to the following performance measures:

- bias in the parameter estimate $({\overset{-}{\beta }}_{T}-\beta_{T},\text{where}{\overset{-}{\beta }}_{T}=\frac{1}{10000}\sum_{j=1}^{10000}{\hat{\beta }}_{Tj}),$
- empirical standard error $(EmpSE=\sqrt{\frac{1}{9999}{\sum_{j=1}^{10000}\left({\hat{\beta }}_{Tj}-{\overset{-}{\beta }}_{T}\right)}^{2}}),$
- model-based standard error $(ModSE=\sqrt{\frac{1}{10000}\sum_{j=1}^{10000}\hat{Var}({\hat{\beta }}_{Tj})}),$
- relative percent error in the model-based standard error $(100(\frac{ModSE}{EmpSE}-1)),$
- coverage (the percentage of 95% confidence intervals for *β_T_* that contain the true value *β_T_*), and
- type I error/power (the percentage of tests of *β_T_* = 0 that are rejected at the 5% level).

Monte Carlo standard errors were calculated for each performance measure based on equations presented else-where.^16^

### 2.3 Estimating treatment effects when only some stratification errors are discovered

To compare the performance of different methods of adjusting for stratification variables when only some stratification errors are discovered, the simulation study described in Section 2.2 was repeated under 2 error discovery patterns. First, stratification errors were discovered at random with probability 0.5, independent of treatment group, as would be expected in blinded trials. Second, the overall probability of discovering an error was kept at 0.5 but errors were three times more likely to be discovered in the intervention arm (ie, the probability of discovering an error was 0.25 in the control arm and 0.75 in the intervention arm). This scenario is plausible in unblinded trials, for example in trials that compare intensive interventions to usual care and thus involve substantially more contact with intervention participants. A total of 32 additional simulation scenarios were therefore considered (2 treatment effects × 2 covariate effects × 4 stratification error patterns × 2 stratification error discovery patterns). A linear regression model withmain effects for the randomised treatment group and *W* (adjusted for the updated strata) was included as an additional method of analysis. Although the true strata are unknown here and hence adjusting for *X* is not possible in practice, this adjustment method was still included in the simulation study for reference.

### 2.4 Estimating treatment by covariate interaction effects

To explore the impact of stratification errors on treatment-by-covariate interaction tests, outcomes were generated from model (1) with *α* = 0, *β_X_* = 1 and (*β_T_, β_TX_*) = (0.2,0), (0.1,0.2) or (0,0.4). The chosen combinations for (*β_T_, β_TX_*) produce treatment effects of 0.2, 0.1 or 0 in stratum *X* = 0 and 0.2, 0.3 or 0.4 in stratum *X* =1, and hence the marginal treatment effect (here, the average treatment effect across the two strata) was fixed at 0.2 across all scenarios. The latter two combinations of (*β_T_, β_TX_*) result in 35% and 88% power, respectively, to detect an interaction effect with a fixed sample size of 1000 (based on a two-way ANOVA with α=0.05). Stratification error patterns varied as before. Errors were either all discovered, discovered at random with probability 0.5 independent of treatment group, or discovered at random with probability 0.25 in the control arm and 0.75 in the intervention arm. This resulted in a total of 36 simulation scenarios (3 combinations for (*β_T_, β_TX_*) × 4 stratification error patterns × 3 stratification error discovery patterns).

The primary estimand of interest was *E*[*Y*(1)-*Y*(0)|*X* = 1] - *E*[*Y*(1) -*Y*(0)|*X* = 0], the expected difference in potential outcomes between the intervention and control arms for stratum *X* = 1 vs *X* = 0, with true value *β_TX_* as used in the data generation process. The data were analysed using a linear regression model with main effects for the randomised treatment group and either *Z*, *X* or *W*, and an interaction between treatment group and either *Z*, *X* or *W*, respectively. In scenarios where all errors were discovered, *W* = *X* and hence only an analysis based on *X* was considered. The performance measures of interest for *β_TX_* were the bias in the parameter estimate, the empirical SE, the relative percent error in the model-based SE, coverage, type I error and power (as defined in Section 2.2 for *β_T_*). Bias in the parameter estimate and coverage were also of interest for *β_T_*, given its role in estimating treatment effects within subgroups defined by the covariate (since the treatment effect = *β_T_* in stratum *X* = 0 and *β_T_* + *β_TX_* in stratum *X* = 1).

A secondary estimand of interest was the marginal treatment effect *E*[*Y*(1) − *Y*(0)], with true value 0.2. This was estimated from a linear regression model with main effects for the randomised treatment group and either no additional covariates,or *Z*, *X* or *W*. No interaction term was included in the model, since interaction terms are rarely included in the primary analysis of trial outcomes^7,9^ and hence it is of interest to understand how the different adjustment approaches perform when an underlying interaction is ignored.

### 2.5 Sensitivity of results to sample size and covariate prevalence

To explore the sensitivity of our simulation results to the chosen sample size and covariate prevalence, we repeated a subset of simulations (see Table 1 for details) with a sample size of 200 instead of 1000 or a covariate prevalence of 0.75 instead of 0.5. In each case, the stratification error rate was set to 20% to highlight any differences between methods and only some errors were discovered to allow the performance of the updated strata to be studied.

## 3 Simulation Results

### 3.1 Effect of stratification errors on the correlation between treatment groups

**Figure 1:**
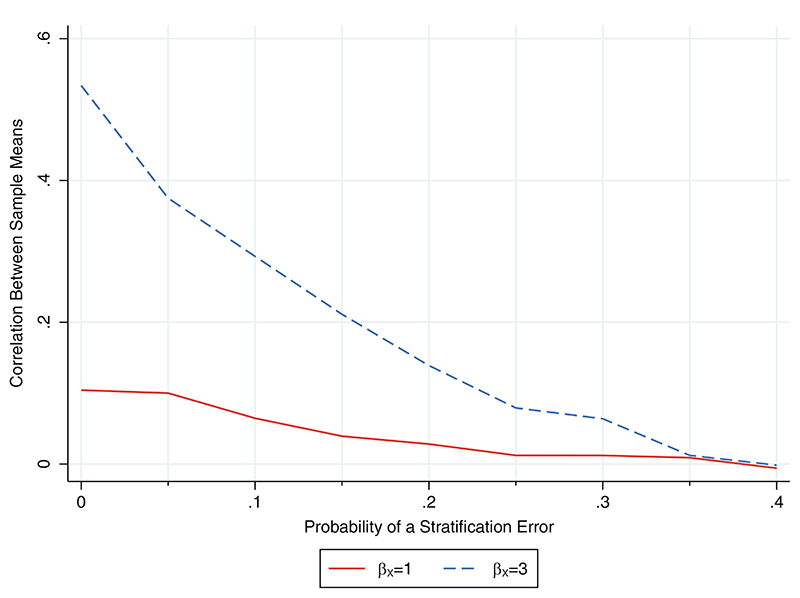
Relationship between the probability of a stratification error and the correlation between the sample means in the intervention and control groups following stratified randomisation based on a covariate with a strong (*β_X_* = 1) or very strong (*β_X_* = 3) relationship with the outcome. The Monte Carlo SE for the Fisher z-transformation of the correlation was 0.01 in each scenario.

When there were no stratification errors, the correlation between the sample means in the two treatment groups was 0.10 for a strong covariate effect (*β_X_* = 1) and 0.53 for a very strong covariate effect (*β_X_* = 3). The correlation decreased as the probability of a stratification error increased and was approximately zero with 40% errors, irrespective of the strength of the covariate effect (Figure 1). This relationship suggests that the problems associated with performing an unadjusted analysis will reduce as stratification errors increase but are unlikely to disappear for realistic error rates.

### 3.2 Estimating treatment effects when all stratification errors are discovered

All methods of analysis (unadjusted, adjusted for the randomisation strata and adjusted for the true strata) produced unbiased treatment effect estimates across all scenarios (results not shown). However, the methods differed according to other performance measures. In the reference setting when there were no stratification errors, the unadjusted analysis performed poorly as expected, producing model-based standard errors that were too large, coverage rates that were too high, type I error rates that were too low and reduced power compared to the adjusted analysis methods. Problems worsened as the covariate effect increased. In contrast, both adjusted analysis methods performed well on all performance measures (see Supplementary Table S1).

**Figure 2:**
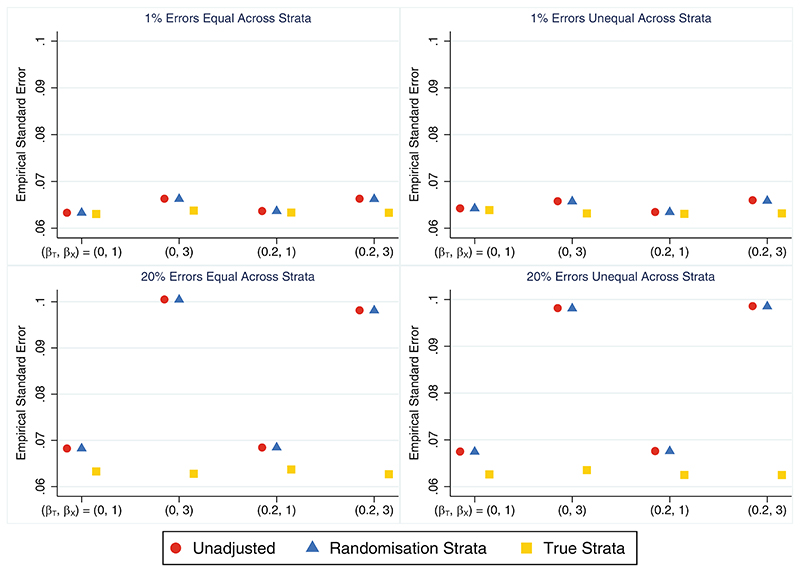
Empirical SE across simulation scenarios comparing an unadjusted analysis, adjusting for the randomisation strata and adjusting for the true strata (which matches the updated strata in this setting) when the treatment effect is of interest and all stratification errors are discovered. The maximum Monte Carlo SE across all methods and scenarios was 0.0007.

When stratification errors occurred, the unadjusted analysis still performed poorly, albeit to a lesser extent when the error rate was high compared to low, and hence the correlation induced by stratification was reduced (as shown in Section 3.1).In comparison to the unadjusted analysis, the models adjusting for the randomisation strata or the true strata both performed well overall, although adjusting for the true strata was preferable to the randomisation strata in some settings. With few stratification errors (1% error rate), there was little difference in performance measures between models adjusting for the randomisation strata or the true strata. Adjusting for the true strata produced marginally smaller empirical standard errors (Figure 2) and was slightly more powerful but only when the covariate effect was very strong (~3% increase in power when *β_X_* = 3; see Table 2). When the stratification error rate was high (20%) and the covariate effect was very strong, adjusting for the true strata produced substantially smaller empirical SEs (Figure 2) and provided a much more powerful analysis (Table 2) compared to adjusting for the randomisation strata (∼36% increase in power). Both adjusted analysis methods performed similarly in terms of the relative percent error in themodel-based SE (Supplementary Figure S1), coverage (Supplementary Figure S2) and type I error (Table 2), and both performed as well or better than the unadjusted analysis across all performance measures. The size of the treatment effect and the distribution of errors across strata had little impact on the simulation results for any analysis method.

### 3.3 Estimating treatment effects when only some stratification errors are discovered

**Figure 3:**
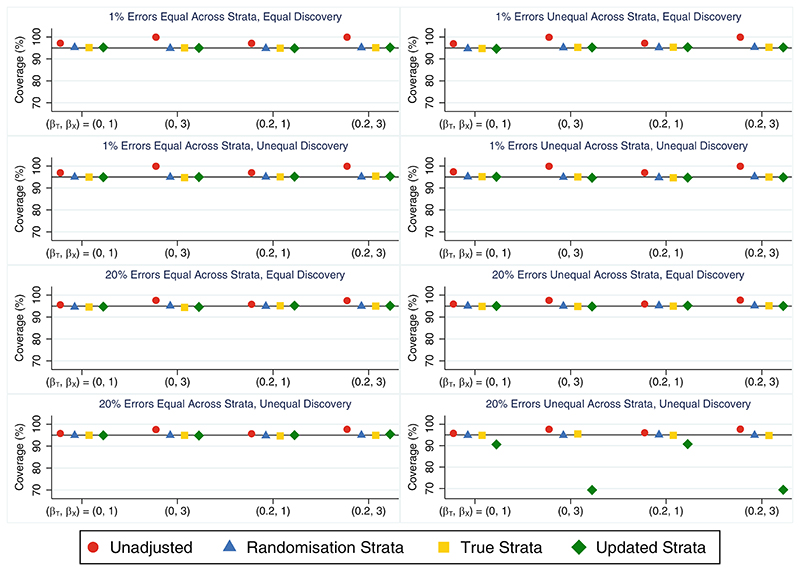
Coverage rates across simulation scenarios compared to the nominal rate of 95% comparing an unadjusted analysis, adjusting for the randomisation strata, adjusting for the true strata and adjusting for the updated strata when the treatment effect is of interest and only some stratification errors are discovered either equally or unequally across treatment groups. The maximum Monte Carlo SE across all methods and scenarios was 0.46.

The unadjusted analysis produced unbiased treatment effect estimates but performed poorly on other performance measures in all scenarios where only some stratification errors were discovered, consistent with the scenarios where all stratification errors were discovered (as expected, since this method is unaffected by whether errors are discovered or not). Adjusting for the true strata was the best performing approach, consistently producing unbiased treatment effect estimates (Supplementary Figure S3) with the smallest empirical standard errors (Supplementary Figure S4) and providing the greatest power (Table 3). As this method cannot be used in practice when some errors go undiscovered, the next best approach was adjusting for the updated strata. This method also produced unbiased treatment effect estimates and had advantages over adjusting for the randomisation strata in terms of empirical standard errors and power across most scenarios. The only exception was when errors were unequal across strata and error discovery was unequal across treatment groups, in which case adjusting for the updated strata produced biased treatment effect estimates (Supplementary Figure S3). This led to poor coverage (Figure 3), inflated type I error rates and reduced power (Table 3) when the error rate was high, and problems worsened as the covariate effect increased. Adjusting for the randomisation strata was preferable in this setting of unequal errors across strata and unequal error discovery across treatment groups, producing unbiased treatment effect estimates and type I error and coverage rates close to nominal levels. Adjusting for the randomisation strata, true strata and updated strata all performed similarly in terms of the relative percent error in the model-based SE (Supplementary Figure S5) with values close to zero in all scenarios. They also performed similarly in terms of coverage (Figure 3) and type I error (Table 3), with values close to nominal levels except in those scenarios where adjusting for the updated strata was biased. Only the analysis based on the updated strata was affected by the distribution of stratification errors across strata or the pattern of error discovery across treatment groups, while the size of the treatment effect had little impact on the results for all analysis methods.

### 3.4 Estimating treatment by covariate interaction effects

**Figure 4:**
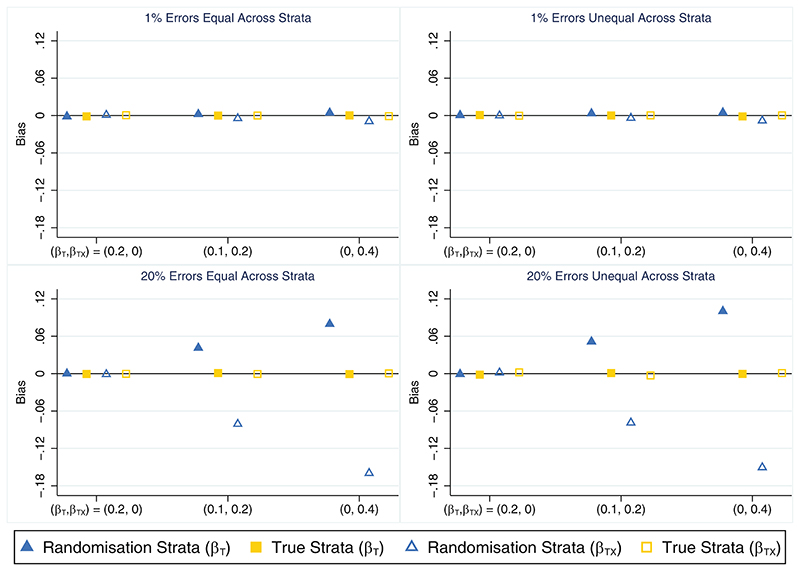
Bias in the treatment (*β_T_*) and interaction (*β_TX_*) parameter estimates across simulation scenarios comparing an analysis based on the randomisation strata and the true strata (which matches the updated strata in this setting) when the treatment by covariate interaction effect is of interest, the covariate effect is strong (*β_X_* = 1) and all stratification errors are discovered. The maximum Monte Carlo SE across all parameters, methods and scenarios was 0.001.

When all stratification errors were discovered, using the true strata to test for an interaction effect performed well on all measures across all scenarios. This method consistently produced unbiased parameter estimates (Figure 4), and type I error (Table 4) and coverage rates (Supplementary Figure S6) that were close to nominal levels. It also produced slightly smaller empirical standard errors for the interaction term (results not shown) and had greater power to detect the interaction effect (Table 4) compared with an analysis using the randomisation strata, especially when stratification errors were common (power ~21% and 48% higher using the true strata when *β_TX_* = 0.2 and 0.4, respectively). In contrast, using the randomisation strata to test for the presence of a treatment-by-covariate interaction effect produced substantially biased estimates of both the treatment (overestimated) and interaction (underestimated) parameters from the data generation model when an interaction effect was present and the error rate was high (Figure 4), which led to lower than nominal coverage rates for both parameters (Supplementary Figure S6). However, this method produced unbiased parameter estimates (Figure 4) and type I errors that were close to the nominal level (Table 4) in the absence of an interaction. The relative percent error in the model-based SE for the interaction term was small for both analysis methods in all scenarios (maximum error=2.3%; results not shown) and the distribution of errors across strata had little impact on the results for either method of analysis. When the analysis model did not include an interaction effect and was used to estimate the marginal treatment effect, the different analysis approaches (unadjusted analysis, adjusting for the randomisation strata or adjusting for the true strata) produced unbiased estimates and performed similarly to the setting with no interaction effect (see Section 3.2), with adjustment for the true strata outperforming the randomisation strata (results not shown).

**Figure 5:**
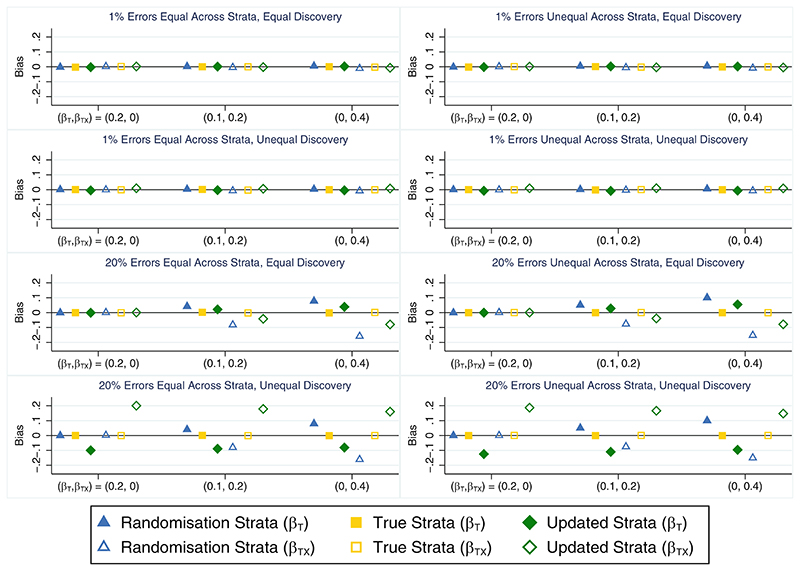
Bias in the treatment (*β_T_*) and interaction (*β_TX_*) parameter estimates across simulation scenarios comparing an analysis based on the randomisation strata, the true strata and the updated strata when the treatment by covariate interaction effect is ofinterest, the covariate effect is strong (*β_X_* = 1) and only some stratification errors are discovered either equally or unequally across treatment groups. The maximum Monte Carlo SE across all parameters, methods and scenarios was 0.001.

When only some stratification errors were discovered, using the updated strata to test for an interaction effect was problematic in some cases. This method produced biased treatment and interaction parameter estimates (Figure 5) leading to lower than nominal coverage rates for both parameters (Supplementary Figure S7) when the error rate was high, except in scenarios where there was no interaction and the errors were discovered equally between the treatment groups. This method also suffered from inflated type I error rates when stratification errors were common and more errors were discovered in one treatment group (Supplementary Table S2). In comparison, the analysis based on the randomisation strata generally performed worse than the analysis using the updated strata in terms of bias (Figure 5), coverage (Supplementary Figure S7) and power (Supplementary Table S2) when errors were equally likely to be discovered in both treatment groups, but had advantages over using the updated strata in terms of reduced bias and closer to nominal coverage when errors were more frequently discovered in one treatment group. The apparent power gains achieved by using the updated strata in scenarios where error discovery depended on treatment group (Supplementary Table S2) resulted from the positive bias in the interaction effect estimates and hence do not demonstrate an advantage of this analysis approach. The relative percent error in the model-based SE for the interaction term was small for all methods across all scenarios (maximum error = 1.8%; data not shown). The analysis based on the randomisation strata produced the largest empirical standard errors for the interaction term, followed by the updated strata (results not shown). Only the ideal but unachievable approach of testing for an interaction using the true strata did well across all performance measures and all scenarios. When the analysis model did not include an interaction term and was used to estimate the marginal treatment effect, the relative performance of the different adjustment approaches was generally similar to the setting with no interaction effect (see Section 3.3), with the updated strata outperforming the randomisation strata except when errors were unequal across strata and error discovery was unequal across treatment groups. The problems with adjusting for the updated strata in this specific setting were accentuated by the presence of an interaction effect (results not shown).

### 3.5 Sensitivity of results to sample size and covariate prevalence

Reducing the sample size from 1000 to 200 resulted in larger empirical standard errors and reduced power across all methods as expected (results not shown). It had little impact on analyses based on the true strata otherwise and little impact on bias for any method. In contrast, the smaller sample size reduced but did not eliminate the undercoverage observed when estimating treatment effects using the updated strata (Supplementary Figure S8), and when estimating treatment by covariate interaction effects using either the randomisation strata or the updated strata (Supplementary Figure S9).

Increasing the covariate prevalence from 0.5 to 0.75 introduced bias and undercoverage into the treatment effect esti-mate based on the updated strata in the setting where errors were equal across strata but error discovery was unequal across treatment groups (Supplementary Figure S8) and increased the magnitude of the bias and undercoverage observed for this method in other settings (Supplementary Figures S8 and S9). Similarly, bias and coverage problems increased when estimating treatment by covariate interaction effects using the randomisation strata (Supplementary Figure S9). Analyses based on the true strata were largely unaffected by the covariate prevalence.

## 4 Example: The Optimise Randomised Trial

To illustrate how different methods of adjusting for stratification variables perform in a real trial affected by stratification errors, we consider data from the OPTIMISE trial.^22^ OPTIMISE was a randomised controlled trial investigating the effect of a dietary and lifestyle intervention compared with standard antenatal care in 641 pregnant women with healthy weight (body mass index in the range 18.5-24.9 kg/m^2^ at the first antenatal appointment). Randomisation was performed by highly trained research staff within a web-based system using 1:1 allocation and randomly permuted blocks of size 4 within strata defined by parity (0 vs 1 or more previous pregnancies that reached at least 20weeks’ gestation) as reported by participants at baseline. The women and the research team delivering the intervention were necessarily unblinded to the treatment group allocation and research staff had substantially more contact with women in the intervention arm due to the nature of the intervention.

Following randomisation, obstetric history was obtained from all participants’ medical records, which revealed stratification errors for 23 women (3.6% of trial participants).Based on their medical records, 59% of women had no previous pregnancies. Accessing medical records prior to randomisation to determine the appropriate strata was not possible due to requirements for consent to access this information and the time delay involved in accessing paper records. The errors resulted from participants incorrectly answering the relevant question (“Have you had any pregnancies of 20weeks or more?”) on the trial screening form, rather than data entry errors in the web-based randomisation system. The reasons for this are unclear, since the screening question was relatively straightforward and language difficulties were not a factor for trial participants. Stratification errors were more common in one stratum, affecting only 5 (1%) women with no previous pregnancies compared to 18 (7%) women with a previous pregnancy. However, the discovery of these errors did not appear to depend on treatment group (11 errors in the intervention arm vs 12 in the control arm).

For illustration, the key secondary outcome of infant birthweight was analysed using a linear regression model including the randomised treatment group and either no additional covariates (unadjusted model), the randomisation strata or the updated strata (which likely equals, or very closely matches, the true strata in this setting where medical records were reviewed for all trial participants). The stratification variable was an important predictor of this outcome, since the mean infant birth weight increased by 107 g (95% CI 21–193), or approximately 0.2 standard deviations, for women with a previous pregnancy compared to women without a previous pregnancy. The estimated treatment effects (SEs) were -79.6 g (43.9), -81.1 g (43.6) and-78.3 g (43.7) when adjusting for no covariates, the randomisation strata and the updated strata, respectively. Differences in treatment effect estimates between methods were small as expected from the simulation results, given the stratification errors were balanced across treatment groups and hence all methods are expected to produce unbiased treatment effect estimates. The unadjusted analysis produced the largest SE, consistent with simulation findings. An ad hoc approach to error discovery in OPTIMISE could have feasibly resulted in more errors being discovered in the intervention group through greater contact with these women, leading to larger differences in results depending on the chosen adjustment approach. For instance, if all errors in the intervention group had been discovered but none in the control group, an analysis adjusting for the updated strata (which likely matches the true strata for the intervention group but matches the randomisation strata for the control group) would have produced an estimated treatment effect (SE) of −82.1 g (43.7), which differs somewhat from the estimate of −78.3 g when adjustment was made for the updated strata and (presumably) all errors were discovered in both treatment groups. This result highlights the potential for the chosen method of adjustment to impact trial results.

## 5 Discussion

In this article, we have used simulation studies and an example dataset to explore the impact of misclassification in a stratification variable when analysing continuous outcomes. Building on previous research,^13–15^ we have considered for the first time the realistic setting where only some stratification errors are discovered. Our results show that while stratification errors reduce the correlation induced by stratification, adjustment for the stratification variable in some form remains important. If treatment effect estimation is of interest, adjusting for the randomisation strata is an acceptable approach and the only option if the errors are never discovered, but adjusting for the true strata (if all errors are discovered) or the updated strata (if only some errors are discovered) generally leads to gains in power. Caution is needed in settings where the discovery of stratification errors may depend on treatment group, since adjusting for the updated strata can introduce bias in this case. These findings apply whether the treatment effect is constant or varies across strata. If treatment-by-covariate interactions and subgroup effects are of interest, only an analysis based on the true strata consistently produces unbiased parameter estimates. These findings highlight the importance of identifying and documenting any stratification errors that do occur, though there is always a risk that some errors will go undiscovered.

With few stratification errors, our simulations revealed little difference in performance between the different adjustment approaches and hence it is unlikely to matter which covariate is used for adjustment (the randomisation strata, true strata or updated strata) in most practical settings. Reassuringly, our results therefore raise no concerns over previous trial results based on any adjustment strategy, provided the error rate was low. Substantial differences between adjustment approaches were seen when the error rate was high, however, and the chosen approach has the potential to influence trial conclusions in this case. Our view is that the best performing method for handling stratification errors should be pre-specified for the analysis, regardless of whether the trial ends up being affected by many errors or only one, and we provide recommendations regarding which methods to pre-specify in Section 6.

Previous work on measurement error has shown that misclassification in a binary predictor variable leads to biased regression coefficient estimates for that predictor and possibly other predictors in a linear regression setting.^23^ We therefore expect regression coefficients to be biased for the stratification variable when stratification errors occur, however the main concern in a clinical trial is whether these errors lead to biased treatment effect estimates. Others have shown theoretically under a linear regression model that the treatment effect estimate is unbiased whether adjustment is made for the randomisation strata or the true strata^13^ and our simulation results are consistent with these theoretical findings. Interestingly, we found that when some errors go undiscovered, adjusting for the updated strata can introduce bias into the treatment effect estimate in the setting where error discovery depends on treatment group, which resulted in problems with other performance measures. The reason for this bias can be seen by considering the extreme case where all errors are discovered in the intervention group (ie, *W_i_* = *X_i_* when *T_i_* = 1) but none are discovered in the control group (ie, *W_i_* = *Z_i_* when *T_i_* = 0). Adjusting for the updated strata thus estimates *E*[*Y*(1)|*W*]-*E*[*Y*(0)|*W*] = *E*[*Y*(1)|*X*]-*E*[*Y*(0)|*Z*], which is biased in general for the adjusted estimand of interest *E*[*Y*(1)|*X*]-*E*[*Y*(0)|*X*]. Such bias is highly unlikely to occur in blinded trials and is most plausible in unblinded trials involving more contact with intervention participants, thus providing more opportunity for any stratification errors to be discovered in this treatment group.

Our simulations confirm previous findings that failing to adjust for stratification variables by performing an unadjusted analysis leads to biased standard errors and overcoverage, while adjusting for the randomisation strata avoids these issues.^10^ Perhaps surprisingly, our simulations show that in the presence of stratification errors, such problems do not occur when adjusting for the true strata rather than the randomisation strata. Intuitively, this occurs because the randomisation was based on a misclassified covariate and by adjusting for the true covariate, the correlation induced by stratification is accounted for. More formally, biased standard errors and overcoverage are expected when there is restricted (eg, blocked) randomisation based on *Z* but no adjustment for *Z* and *P*(*Y | Z,**V***) ≠ *P*(*Y | **V***), where ***V*** is a vector of covariates for adjustment. In the case of stratification errors, where the outcome *Y* depends on the true strata *X* but not the randomisation strata *Z* except through the relationship *Z* = *X + error*, *P*(*Y | Z,X*) = *P*(*Y | X*) and hence adjustment for *X* is sufficient to avoid biased standard errors and overcoverage.^24^

A limitation of our study is that we only considered continuous outcomes. Binary, count and time-to-event outcomes analysed via logistic regression, log Poisson regression and Cox proportional hazardsmodels, respectively, have been studied previously.^13,14^ In each case, treatment effect estimation was of interest and all stratification errors were discovered. For each type of outcome, the type I error rate was maintained at the nominal level whether adjustment was made for the randomisation strata (provided allowance was made for overdispersion in the case of count outcomes) or the true strata, while adjusting for the true strata was more powerful, consistent with our findings for continuous outcomes. No bias was observed in the estimated treatment effects, except in the case of binary outcomes where adjusting for the randomisation strata produced biased estimates of the conditional log-odds ratio (due to the change in estimand being estimated by models adjusting for different covariates that is a known issue with logistic regression^25^). We are not aware of any studies considering non-continuous outcomes in settings where treatment-by-covariate interaction effects are of interest or only some stratification errors are discovered, and these are potential topics for future research. Our work could also be extended in several other ways. First, the stratification variable could have more than two categories and be included in tthe analysis using random effects, as is generally recommended when stratifying by centre inmulticentre trials.^26^ Second, unequal treatment allocation (eg, 2:1 randomisation) could be considered, which may necessitate both the inclusion of treatment-by-covariate interactions in the model for estimating treatment effects and use of an alternate variance estimator to avoid inflated type I errors.^27^ Third, misclassification in balancing variables for other forms of covariate-adaptive randomisation, such as minimisation, could be investigated in settings where only some errors are discovered, building on previous work by others.^14,15^ Finally, many trials use multiple stratification variables^1,2^ and the impact of varying levels of misclassification and error discovery across stratification variables could be explored.

## 6 Recommendations For Practice

Stratification errors can occur in any trial that uses stratified randomisation. We encourage researchers to consider the possibility of stratification errors at all stages of the trial and provide 10 general recommendations for addressing these errors during the design, conduct, analysis and reporting of the trial in Table 5. Most importantly for the trial statistician, we recommend pre-specifying how stratification errors will be addressed in the primary analysis and any planned secondary or subgroup analyses in the statistical analysis plan (Recommendation 2) to help avoid the risk of p-hacking.^28^ The findings from our simulation study and previous research^13–15^ yield important insights into the best analytic methods to pre-specify in the statistical analysis plan and provide the basis for Recommendations 2a and b, as detailed below. Since researchers will likely never know whether they have identified all stratification errors or not, our recommendations focus on choosing between the randomisation strata and the updated strata, rather than the ideal but possibly unobtainable true strata.

In most trials, the discovery of stratification errors is unlikely to be related to treatment group. For example, in blinded trials the research staff typically have similar amounts of contact with participants in each group, providing similar opportunities for errors to be discovered in each group. In this case, we recommend adjusting for the updated strata in the primary analysis for all trial outcomes (Recommendation 2a) due to its power advantages. Adjusting for the randomisation strata should be considered in a sensitivity analysis, at least for the primary outcome, to explore the impact of any stratification errors on the trial results. If subgroup analyses by the stratification variable are planned, we recommend using the updated strata to define the subgroups and perform the interaction test in order to minimise any bias in the subgroup effect estimates.

An exception to these recommendations should be made when the discovery of stratification errors could plausibly be related to treatment group. This can occur, for example, in unblinded trials involving substantially greater contact with trial participants assigned to one treatment arm and hence providing a greater opportunity for stratification errors to be discovered among these participants. In this setting, we recommend adjusting for the randomisation strata in the primary analysis for all trial outcomes (Recommendation 2b) to avoid the risk of introducing bias into the treatment effect estimates. Adjusting for the updated strata should be performed in a sensitivity analysis, at least for the primary outcome, to explore the impact of any stratification errors on the trial results, while acknowledging the results of this sensitivity analysis could be biased. The risk of bias can be judged by examining and reporting the number of stratification errors that were identified by treatment group, with bias expected when the discovery of stratification errors is more common in one group. If subgroup analyses by the stratification variable are planned, we recommend using the randomisation strata to define the subgroups and perform the interaction test. Although this approach will likely be biased and this should be acknowledged, less bias is expected compared to using the updated strata in this setting.

Existing analysis guidelines state that ‘if one or more factors are used to stratify the design, it is appropriate to account for those factors in the analysis’^7^ and ‘the factors that are the basis of stratification should normally be included as covariates or stratification variables in the primary outcome model’,^9^ but do not address the problem of stratification errors. Our recommendations add clarification regarding stratification errors and may be helpful for informing future updates of these guidelines.

## 7 Conclusion

Stratification is a useful tool for achieving balanced treatment groups on important baseline covariates, but greater attention should be given to the risk of stratification errors and the implications these errors have for the trial analysis. In most trials, the discovery of stratification errors is unlikely to depend on treatment group and hence using the updated strata, where the errors that have been discovered are corrected, is preferable to using the randomisation strata for adjustment and performing subgroup analyses. In trials where the discovery of stratification errors could plausibly depend on treatment group, analyses should be based on the randomisation strata. Given the potential for stratification errors to introduce bias into treatment and subgroup effect estimates, greater transparency is needed in the reporting of stratification errors and how they were addressed in the analysis.

## Supplementary Material

S1

## Figures and Tables

**Table 1:** Summary of simulation parameters included in each simulation setting.

Simulation setting	Total sample size	Covariate prevalence	α	*β_T_*	*β_X_*	*β_TX_*	Stratification error rate	Errors across strata	Error discovery rate	Error discovery across treatment groups	Number of scenarios
Effect of stratification errors on correlation between treatment groups (Section 2.1)	1000	0.5	0	0	1, 3	0	0%, 5%, 10%, 15%, 20%, 25%, 30%, 35%, 40%	Equal	100%	N/A	18
Estimating treatment effects when all stratification errors are discovered (Section 2.2)	1000	0.5	0	0,0.2	1,3	0	1%, 20%	Equal, Unequal (three times higher in one stratum)	100%	N/A	16
Reference case: Estimating treatment effects when there are no stratification errors (Section 2.2)	1000	0.5	0	0,0.2	1,3	0	0%	N/A	N/A	N/A	4
Estimating treatment effects when only some stratification errors are discovered (Section 2.3)	1000	0.5	0	0,0.2	1,3	0	1%, 20%	Equal, Unequal (three times higher in one stratum)	50%	Equal, Unequal (three times higher in one group)	32
Estimating treatment by covariate interaction effects when all stratification errors are discovered (Section 2.4)	1000	0.5	0	0,0.1, 0.2	1	0.4 (when *β_T_*=0), 0.2 (when *β_T_*=0.1), 0 (when *β_T_*=0.2)	1%, 20%	Equal, Unequal (three times higher in one stratum)	100%	N/A	12
Estimating treatment by covariate interaction effects when only some stratification errors are discovered (Section 2.4)	1000	0.5	0	0,0.1, 0.2	1	0.4 (when *β_T_*=0), 0.2 (when *β_T_*=0.1), 0 (when *β_T_*=0.2)	1%, 20%	Equal, Unequal (three times higher in one stratum)	50%	Equal, Unequal (three times higher in one group)	24
Sensitivity simulations: Estimating treatment effects when only some stratification errors are discovered (Section 2.5)	200,1000	0.5 (when *n* = 200), 0.75 (when *n* = 1000)	0	0	3	0	20%	Equal, Unequal (three times higher in one stratum^a^)	50%	Equal, Unequal (three times higher in one group)	8
Sensitivity simulations: Estimating treatment by covariate interaction effects when only some stratification errors are discovered (Section 2.5)	200,1000	0.5 (when *n* = 200), 0.75 (when *n* = 1000)	0	0	1	0.4	20%	Equal, Unequal (three times higher in one stratum^a^)	50%	Equal, Unequal (three times higher in one group)	8

a When the covariate prevalence was 0.75, the error rate was three times higher in the more common stratum.

**Table 2:** Type I error and power (%) across simulation scenarios comparing an unadjusted analysis, adjusting for the randomisation strata and adjusting for the true strata when the treatment effect is of interest and all stratification errors are discovered.

			Type I error (%) (*β_T_* = 0)			Power (%) (*β_T_* = 0.2)		
Stratification error rate	Errors across strata	Covariate effect (*β_X_*)	Unadjusted	Randomisation strata	True strata^a^	Unadjusted	Randomisation strata	True strata^a^
1%	Equal	1	2.85	4.90	4.85	83.05	88.13	88.40
1%	Equal	3	0.07	4.92	5.12	36.64	85.59	88.80
1%	Unequal	1	3.13	5.38	5.39	82.96	87.89	88.30
1%	Unequal	3	0.05	4.66	5.01	35.94	85.48	88.11
20%	Equal	1	4.21	5.09	4.98	81.80	83.79	88.37
20%	Equal	3	2.74	5.68	4.68	39.81	51.88	88.54
20%	Unequal	1	4.04	4.74	4.71	81.78	83.86	89.10
20%	Unequal	3	2.16	5.00	5.25	40.31	52.89	88.87

*Note*: The maximum Monte Carlo SE across all methods and scenarios was 0.23 for type I error and 0.50 for power.

a Matches the updated strata in this setting where all stratification errors are discovered.

**Table 3:** Type I error and power (%) across simulation scenarios comparing an unadjusted analysis, adjusting for the randomisation strata, adjusting for the true strata and adjusting for the updated strata when the treatment effect is of interest and only some stratification errors are discovered.

Error discovery across treatment groups	Stratification error rate	Errors across strata	Covariate effect (*β_X_*)	Type I Error (%) (*β_T_* = 0)				Power (%) (*β_T_* = 0.2)			
				Unadjusted	Randomisation strata	True strata	Updated strata	Unadjusted	Randomisation strata	True strata	Updated strata
Equal	1%	Equal	1	2.80	4.74	4.86	4.75	82.67	87.79	88.02	87.98
Equal	1%	Equal	3	0.07	5.14	4.95	5.00	35.78	85.27	88.21	86.81
Equal	1%	Unequal	1	3.05	5.32	5.26	5.35	83.53	88.32	88.57	88.41
Equal	1%	Unequal	3	0.11	4.84	4.71	4.78	36.31	85.65	88.21	87.03
Equal	20%	Equal	1	4.41	5.40	5.41	5.25	80.71	83.04	87.99	85.15
Equal	20%	Equal	3	2.36	4.91	5.59	5.39	41.11	52.85	88.84	65.20
Equal	20%	Unequal	1	4.09	4.95	5.16	4.95	81.44	83.48	88.40	85.60
Equal	20%	Unequal	3	2.40	4.99	5.19	5.17	39.08	52.32	88.17	64.53
Unequal	1%	Equal	1	3.05	5.02	5.04	5.07	83.14	88.14	88.73	88.43
Unequal	1%	Equal	3	0.09	5.06	5.24	5.02	36.06	85.95	88.43	87.02
Unequal	1%	Unequal	1	2.63	4.87	4.82	4.89	83.26	88.19	88.40	87.45
Unequal	1%	Unequal	3	0.05	5.09	4.96	5.33	36.81	85.91	88.55	84.70
Unequal	20%	Equal	1	4.25	5.08	5.03	5.05	81.87	83.77	88.28	85.75
Unequal	20%	Equal	3	2.46	5.06	5.06	5.16	41.48	52.54	88.61	65.76
Unequal	20%	Unequal	1	4.26	5.19	5.17	9.43	82.04	83.92	88.62	67.86
Unequal	20%	Unequal	3	2.35	5.18	4.53	30.66	40.00	52.03	88.03	15.04

*Note:* The maximum Monte Carlo SE across all methods and scenarios was 0.46 for type I error and 0.50 for power.

**Table 4:** Type I error and power (%) for the interaction test across simulation scenarios comparing an analysis based on the randomisation strata and the true strata when the treatment by covariate interaction effect is of interest, the covariate effect is strong (*β_X_* = 1) and all stratification errors are discovered.

Stratification error rate	Errors across strata	Type I error (%) (*β_TX_* = 0)		Power (%) (*β_TX_* = 0.2)		Power (%) (*β_TX_* = 0.4)	
		Randomisation strata	True strata^a^	Randomisation strata	True strata^a^	Randomisation Strata	True strata^a^
1%	Equal	5.16	5.08	33.77	34.98	86.45	88.21
1%	Unequal	4.71	5.16	33.61	35.40	86.89	88.61
20%	Equal	4.99	5.23	13.70	35.01	40.08	88.22
20%	Unequal	4.51	5.11	13.23	34.43	41.80	88.93

*Note*: The maximum Monte Carlo standard error across all methods and scenarios was 0.22 for type I error and 0.49 for power.

a Matches the updated strata in this setting where all stratification errors are discovered.

**Table 5:** Recommendations for addressing stratification errors by trial stage in trials using stratified randomisation.

Trial stage	Recommendations
Design	Implement strategies to minimise the risk of stratification errors, such as clearly defining stratification variables, avoiding stratification variables that are likely to be subject to high levels of misclassification, trainingstafftocarefullycheckthestratificationvariablesbeforerandomisingparticipants,anddesigning randomisation systems that are easy to use. Pre-specify how stratification errors will be addressed in the primary analysis and any planned secondary or subgroup analyses in the statistical analysis plan. If the discovery of errors is unlikely to be related to treatment group (eg, in a blinded trial), then (i) adjust for the updated strata^a^ in the primary analysis, (ii) adjust for the randomisation strata^a^ in a sensitivity analysis, and (iii) use the updated strata^a^ to perform any subgroup analyses. If the discovery of errors could plausibly be related to treatment group (eg, in an unblinded trial involving more contact with intervention participants), then (i) adjust for the randomisation strata^a^ in the primary analysis, (ii) adjust for the updated strata^a^ in a sensitivity analysis, and (iii) use the randomisation strata^a^ to perform any subgroup analyses.
Conduct	3. Implement systematic strategies to identify stratification errors across all participants, such as cross-checking stratification variables against alternate data sources (where available).
	4. Thoroughly document any stratification errors that are identified, including how they occurred, to assist with stafftraining and inform the design offuture trials.
	5. Record the updated strata^a^ in a new field in the trial database and leave the randomisation strata^a^ unchanged as a record of how the randomisation was performed.
Analysis	6. Examine the number of stratification errors that were identified overall and broken down by treatment group, stratum and their combination.
	7. Address stratification errors in the planned analyses using the approach(es) pre-specified in the statistical analysis plan.
	8. Consider whether any additional, unplanned analyses should be performed, based on the number and pattern of stratification errors that were identified in the trial (eg, a sensitivity analysis excluding participants affected by stratification errors).
Reporting	9. Reportthenumberofstratificationerrorsthatwereidentifiedbytreatmentgroupandstratum,potentially in a Supplementary Appendix S1, or state that no such errors were identified.
	10. Indicate whether the randomisation strata^a^ or the updated strata^a^ was used in each analysis involving the stratification variables.

The *randomisation strata* are the strata used to perform the randomisation that may be incorrect for some participants. The *updated strata* are the strata based on the best information available at the time of the analysis, where those errors that have been identified are corrected but some errors may remain.

## Data Availability

The simulated data used to generate the findings presented in this article can be generated using the Stata code provided as supplementary material. The data from the OPTIMISE trial example are not publicly available due to privacy restrictions but can be requested by contacting the second author.
