# Supplementary material for: Handling misclassified stratification variables in the analysis of randomised trials with continuous outcomes
Source: Stat Med. 2023 Jun 26;42(19):3529–46. doi: 10.1002/sim.9818 (PMC7614797; doi:10.1002/sim.9818)
Supplement: Supplementary file 1 — Appendix S1. Supporting information. [file SIM-42-3529-s001.pdf]

**Supplementary Appendix**

**Yelland et al. Handling misclassified stratification variables in the analysis of randomised trials with continuous outcomes**

**Supplementary Table S1:** Simulation results comparing an unadjusted analysis and an analysis adjusting for the stratification variable when the treatment effect is of interest and there are no stratification errors

| Treatment Effect ($\beta_T$) | Covariate Effect ($\beta_X$) | Performance Measure | Unadjusted Analysis | Adjusted Analysis* |
|---|---|---|---|---|
| 0 | 1 | Empirical SE | 0.06 | 0.06 |
| | | Relative Error in Model-Based SE (%) | 12.21 | 0.38 |
| | | Coverage (%) | 96.96 | 94.93 |
| | | Type I Error (%) | 3.04 | 5.07 |
| 0 | 3 | Empirical SE | 0.06 | 0.06 |
| | | Relative Error in Model-Based SE (%) | 80.39 | 0.32 |
| | | Coverage (%) | 99.98 | 94.82 |
| | | Type I Error (%) | 0.02 | 5.18 |
| 0.2 | 1 | Empirical SE | 0.06 | 0.06 |
| | | Relative Error in Model-Based SE (%) | 10.97 | -0.71 |
| | | Coverage (%) | 96.84 | 94.57 |
| | | Power (%) | 83.23 | 88.23 |
| 0.2 | 3 | Empirical SE | 0.06 | 0.06 |
| | | Relative Error in Model-Based SE (%) | 80.97 | 0.48 |
| | | Coverage (%) | 99.94 | 95.35 |
| | | Power (%) | 35.91 | 88.47 |

SE = Standard Error

* Adjusted for the randomisation strata or the true strata, as these variables coincide in this setting where there are no stratification errors.

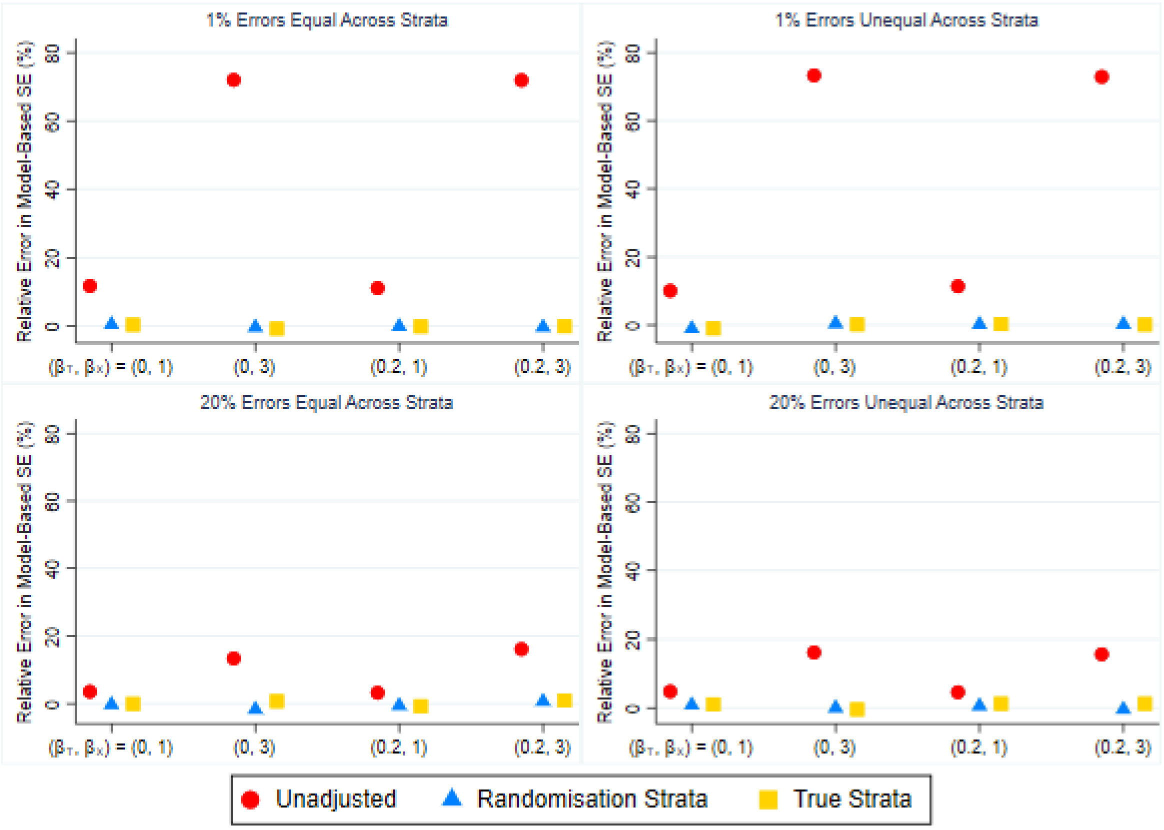


**Supplementary Figure S1:** Relative percent error in the model-based standard error (SE) across simulation scenarios comparing an unadjusted analysis, adjusting for the randomisation strata and adjusting for the true strata (which matches the updated strata in this setting) when the treatment effect is of interest and all stratification errors are discovered. The maximum Monte Carlo standard error across all methods and scenarios was 1.23.

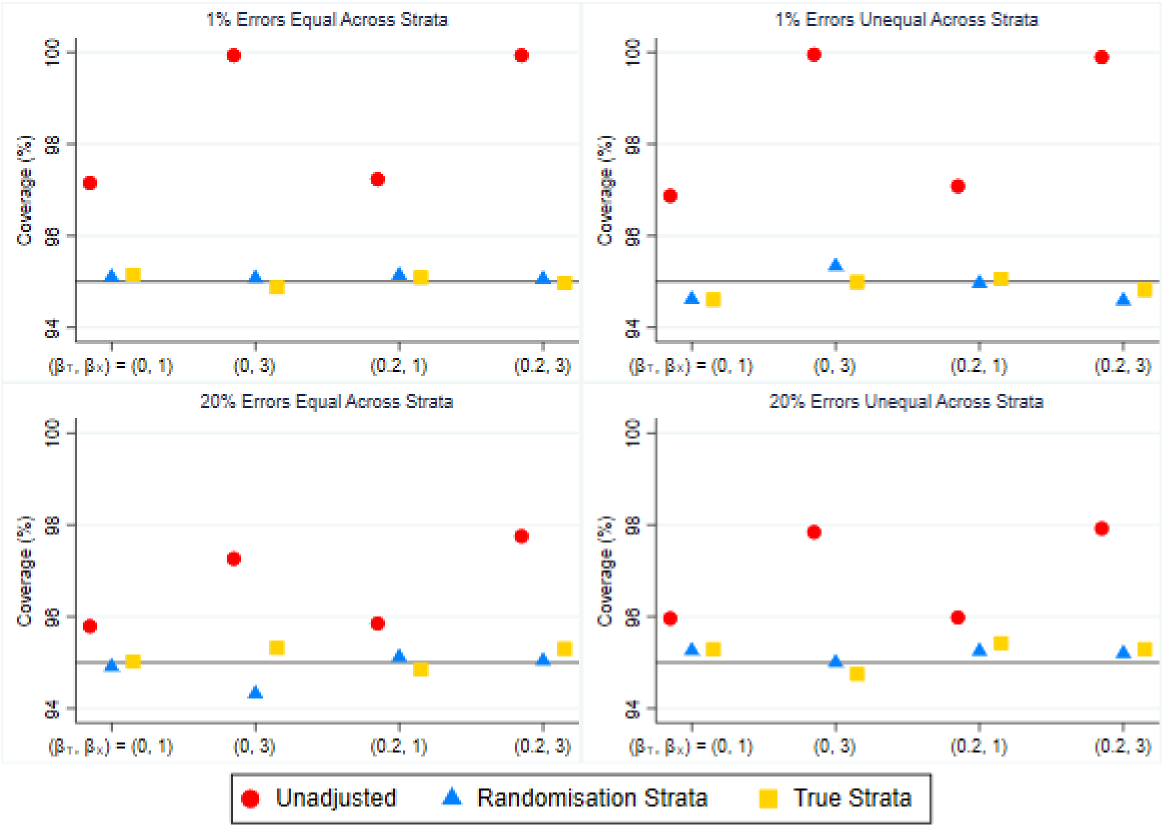


**Supplementary Figure S2:** Coverage rate across simulation scenarios compared to the nominal rate of 95% comparing an unadjusted analysis, adjusting for the randomisation strata and adjusting for the true strata (which matches the updated strata in this setting) when the treatment effect is of interest and all stratification errors are discovered. The maximum Monte Carlo standard error across all methods and scenarios was 0.23.

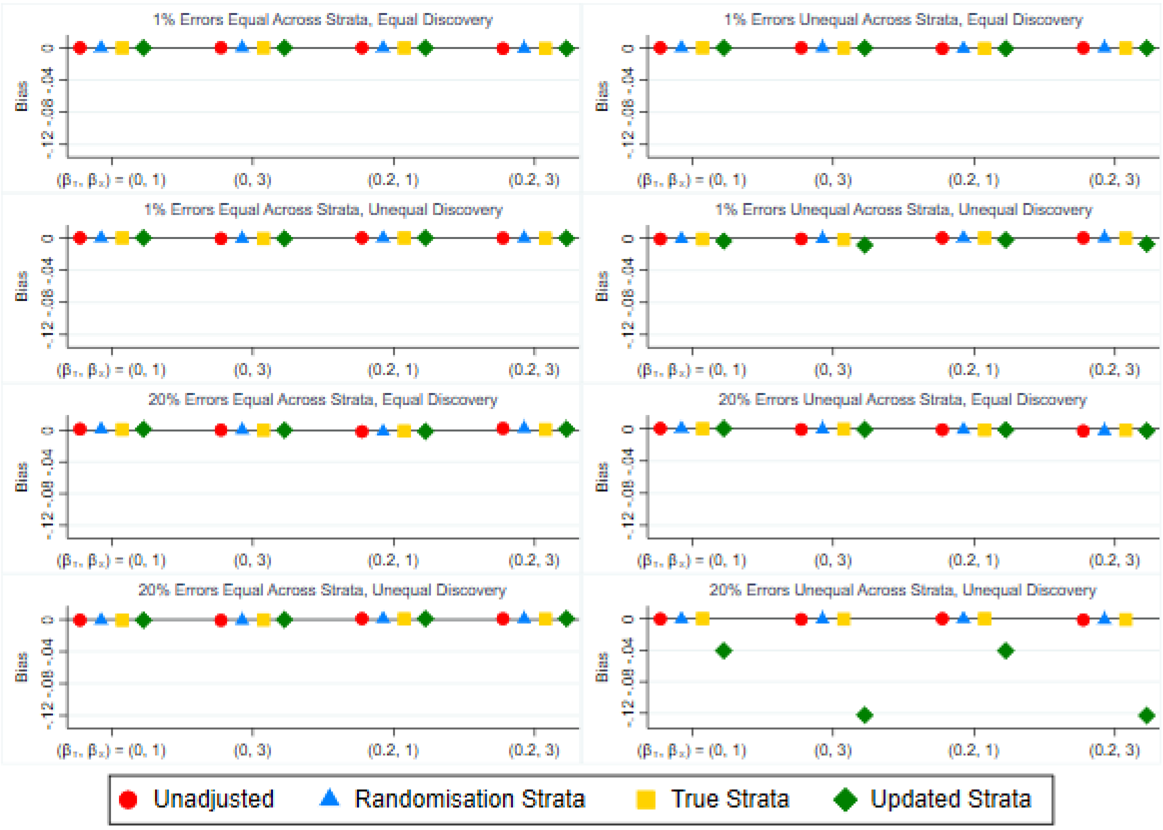


**Supplementary Figure S3:** Bias in the treatment ($\beta_T$) parameter estimate across simulation scenarios comparing an unadjusted analysis, adjusting for the randomisation strata, adjusting for the true strata and adjusting for the updated strata when the treatment effect is of interest and only some stratification errors are discovered either equally or unequally across treatment groups. The maximum Monte Carlo standard error across all methods and scenarios was 0.001.

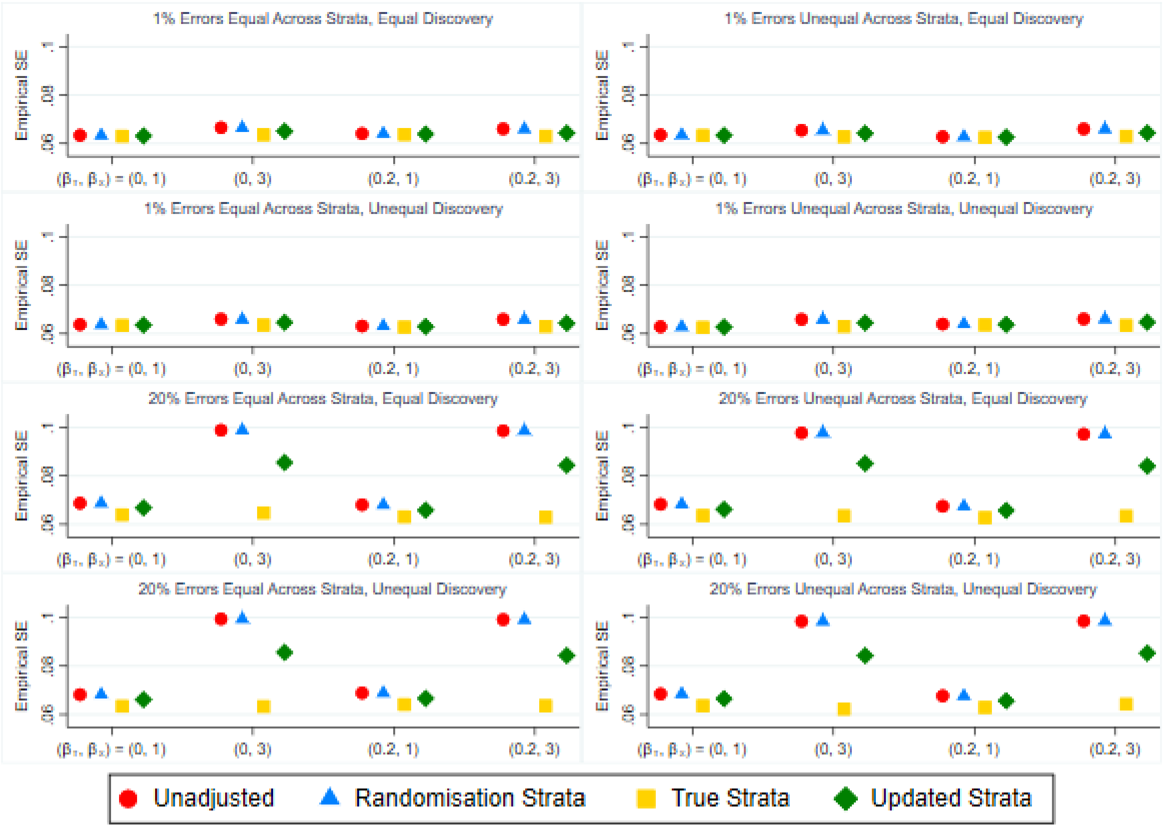


**Supplementary Figure S4:** Empirical standard error (SE) across simulation scenarios comparing an unadjusted analysis, adjusting for the randomisation strata, adjusting for the true strata and adjusting for the updated strata when the treatment effect is of interest and only some stratification errors are discovered either equally or unequally across treatment groups. The maximum Monte Carlo standard error across all methods and scenarios was 0.0007.

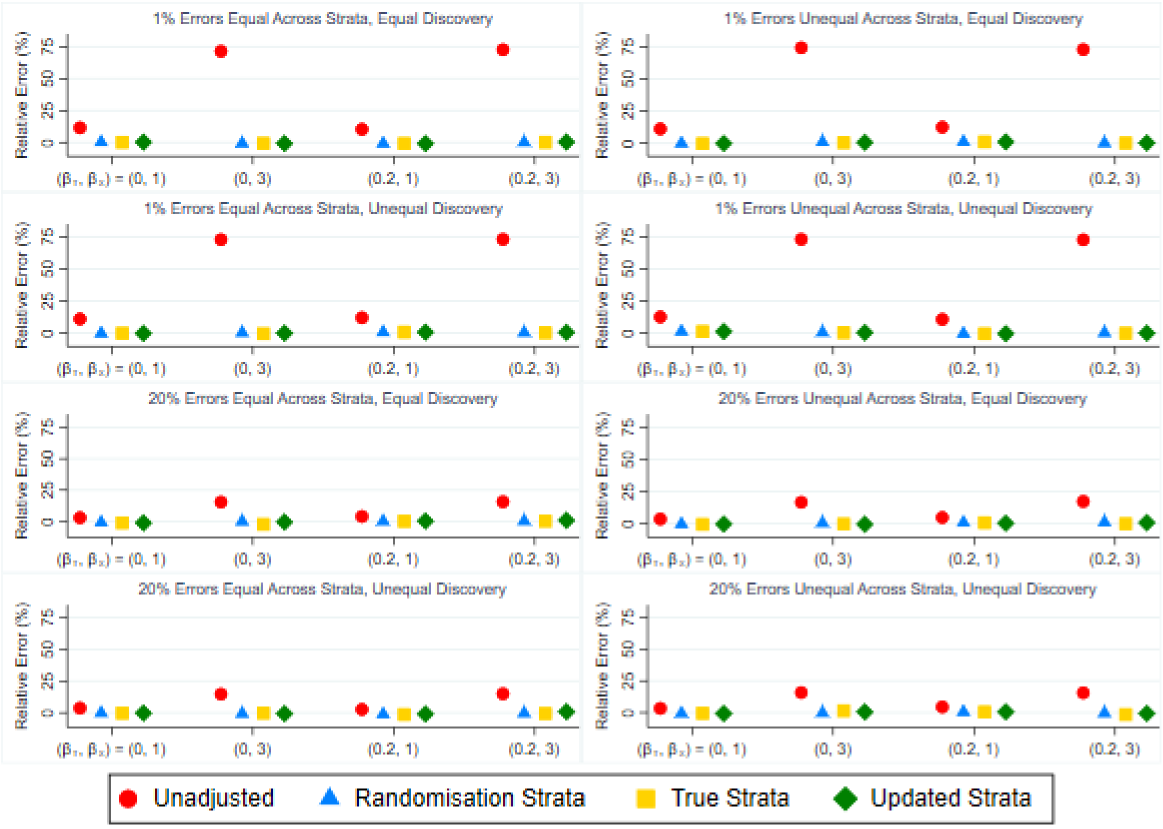


**Supplementary Figure S5:** Relative percent error in the model-based standard error across simulation scenarios comparing an unadjusted analysis, adjusting for the randomisation strata, adjusting for the true strata and adjusting for the updated strata when the treatment effect is of interest and only some stratification errors are discovered either equally or unequally across treatment groups. The maximum Monte Carlo standard error across all methods and scenarios was 1.23.

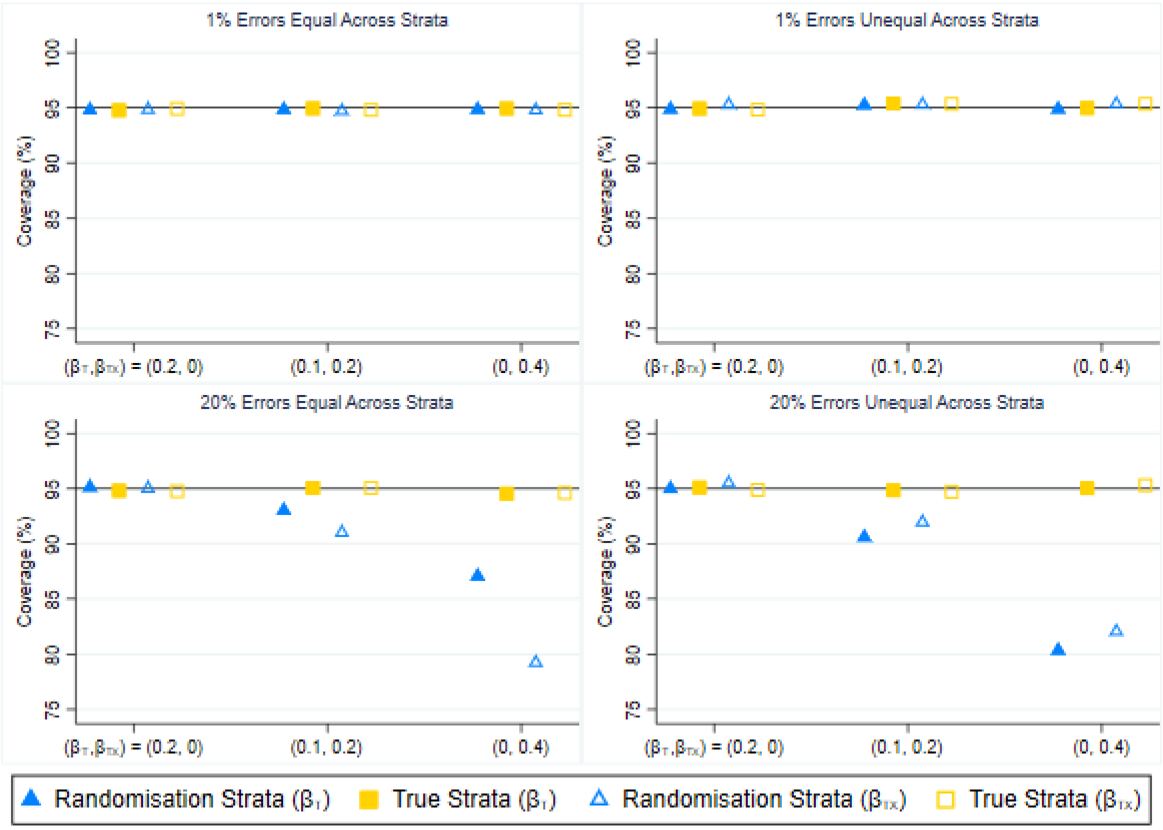


**Supplementary Figure S6:** Coverage rate of 95% confidence interval for the treatment ($\beta_T$) and interaction ($\beta_{TX}$) parameters across simulation scenarios comparing an analysis based on the randomisation strata and the true strata (which matches the updated strata in this setting) when the interaction effect is of interest, the covariate effect is strong ($\beta_X = 1$) and all stratification errors are discovered. The maximum Monte Carlo standard error across all parameters, methods and scenarios was 0.41.

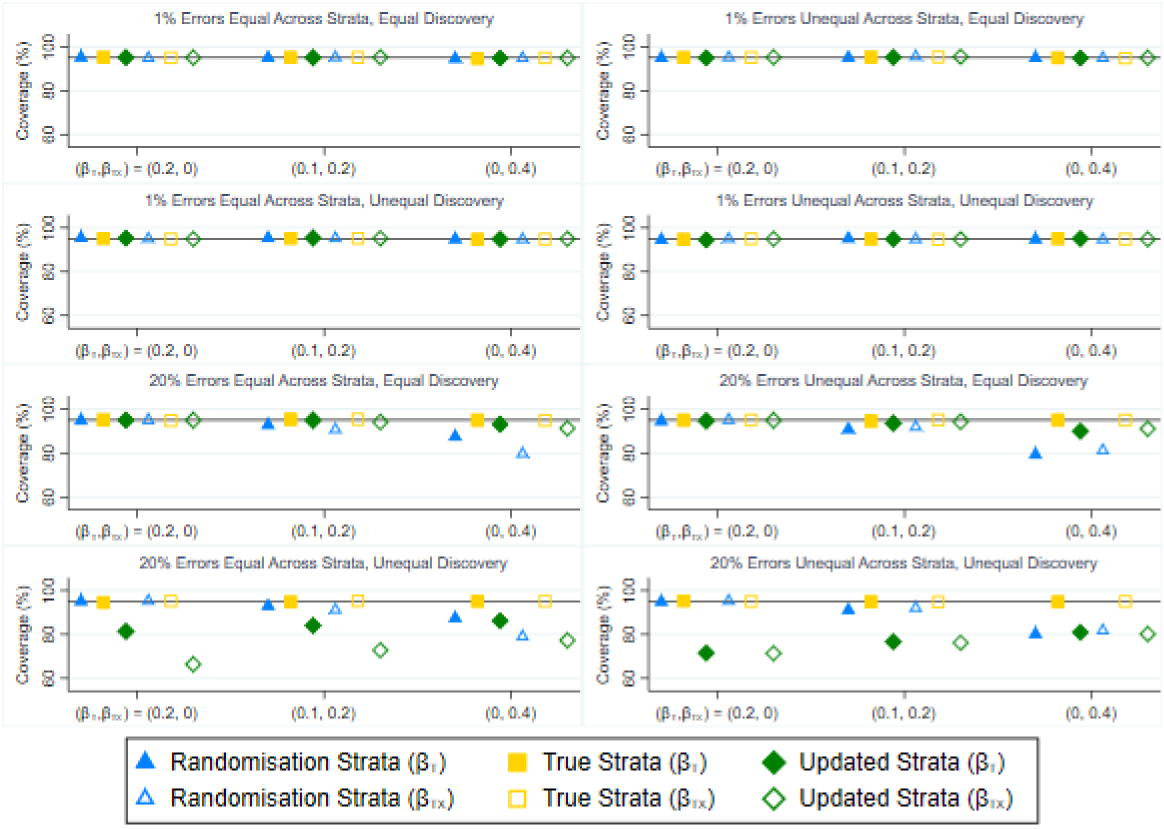


**Supplementary Figure S7:** Coverage rate of 95% confidence interval for the treatment ($\beta_T$) and interaction ($\beta_{TX}$) parameters across simulation scenarios comparing an analysis based on the randomisation strata, the true strata and the updated strata when the interaction effect is of interest, the covariate effect is strong ($\beta_X = 1$) and only some stratification errors are discovered either equally or unequally across treatment groups. The maximum Monte Carlo standard error across all parameters, methods and scenarios was 0.47.

**Supplementary Table S2:** Type I error and power (%) for the interaction test across simulation scenarios comparing an analysis based on the randomisation strata, the true strata and the updated strata when the treatment by covariate interaction effect is of interest, the covariate effect is strong ($\beta_X = 1$) and only some stratification errors are discovered. The maximum Monte Carlo standard error across all methods and scenarios was 0.47 for type I error and 0.49 for power.

| Error Discovery Across Treatment Groups | Stratification Error Rate | Errors Across Strata | Type I Error (%) ($\beta_{TX} = 0$) | | | Power (%) ($\beta_{TX} = 0.2$) | | | Power (%) ($\beta_{TX} = 0.4$) | | |
|---|---|---|---|---|---|---|---|---|---|---|---|
| | | | Randomisation Strata | True Strata | Updated Strata | Randomisation Strata | True Strata | Updated Strata | Randomisation Strata | True Strata | Updated Strata |
| Equal | 1% | Equal | 5.20 | 5.01 | 5.00 | 33.41 | 35.36 | 34.35 | 86.33 | 88.20 | 87.23 |
| Equal | 1% | Unequal | 4.89 | 4.89 | 4.84 | 33.41 | 35.12 | 34.20 | 86.55 | 88.13 | 87.55 |
| Equal | 20% | Equal | 5.05 | 5.19 | 5.06 | 13.49 | 34.35 | 21.73 | 40.35 | 88.29 | 66.10 |
| Equal | 20% | Unequal | 4.97 | 4.95 | 5.13 | 13.84 | 35.48 | 22.71 | 40.92 | 88.54 | 65.99 |
| Unequal | 1% | Equal | 4.99 | 5.09 | 5.17 | 32.96 | 34.83 | 36.96 | 86.89 | 88.61 | 89.61 |
| Unequal | 1% | Unequal | 5.04 | 5.02 | 5.08 | 34.36 | 35.52 | 38.10 | 86.63 | 88.24 | 88.99 |
| Unequal | 20% | Equal | 4.80 | 4.80 | 33.65 | 14.00 | 35.30 | 81.61 | 39.74 | 88.75 | 98.78 |
| Unequal | 20% | Unequal | 4.79 | 5.00 | 28.67 | 13.59 | 35.59 | 78.36 | 41.41 | 88.50 | 98.51 |

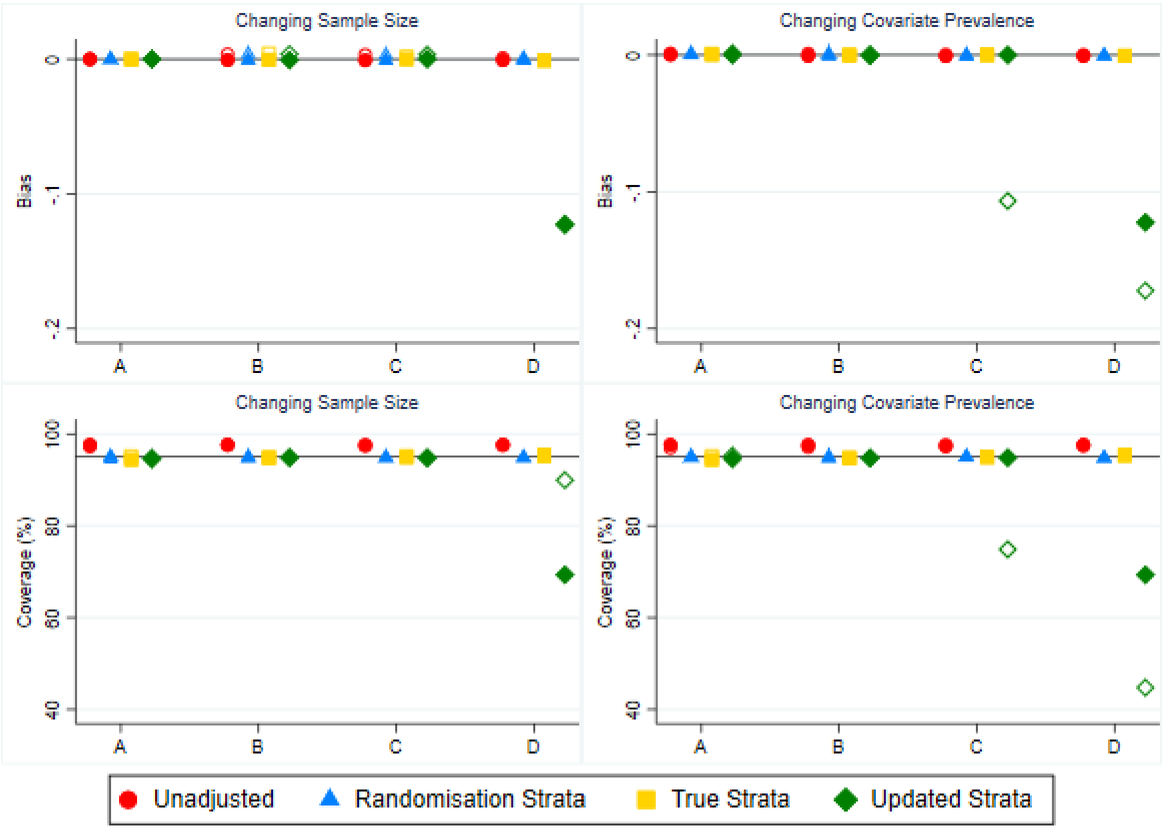


**Supplementary Figure S8:** Bias and coverage rate across simulation scenarios comparing an unadjusted analysis, adjusting for the randomisation strata, adjusting for the true strata and adjusting for the updated strata when the treatment effect is of interest and A) errors are equal across strata and error discovery is equal across treatment groups; B) errors are unequal across strata and error discovery is equal across treatment groups; C) errors are equal across strata and error discovery is unequal across treatment groups; and D) errors are unequal across strata and error discovery is unequal across treatment groups. Solid symbols are original simulation results and open symbols are results after changing the sample size from 1000 to 200, or changing the covariate prevalence from 0.5 to 0.75 (if only a solid symbol is observed, results coincide).

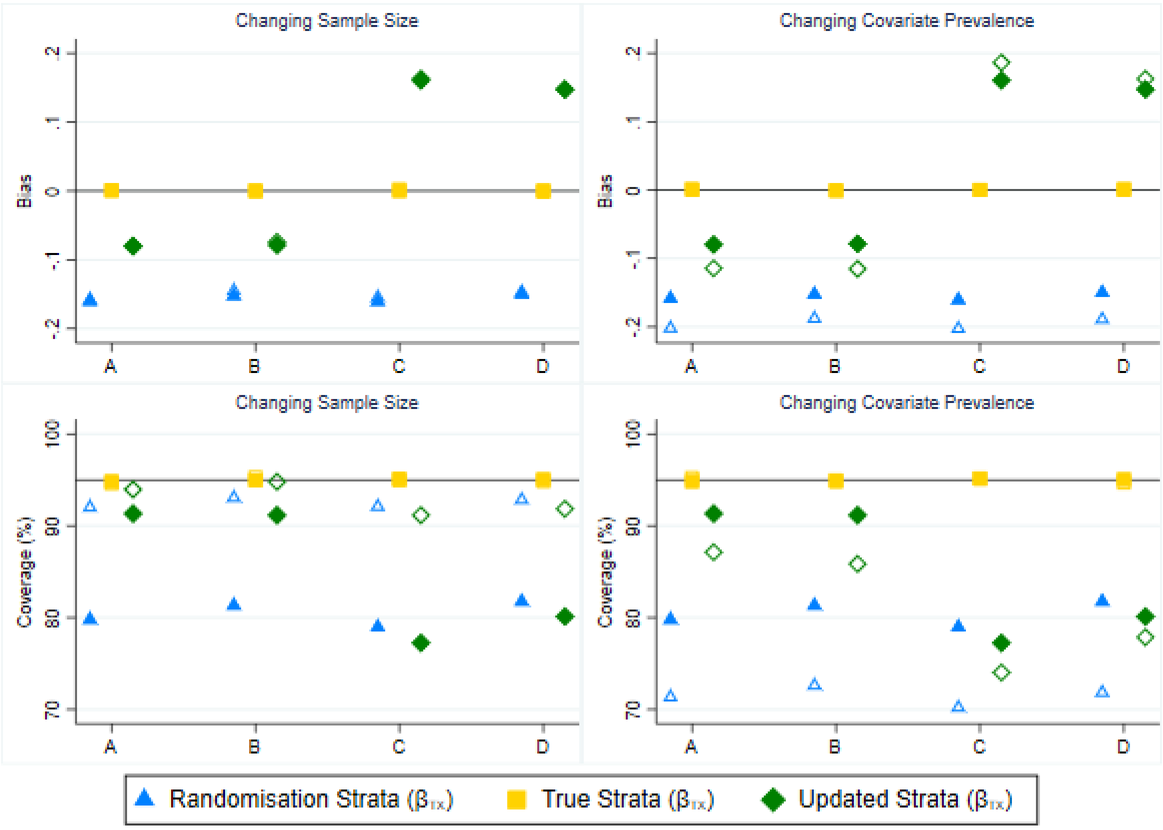


**Supplementary Figure S9:** Bias and coverage rate for the interaction ($\beta_{TX}$) parameter across simulation scenarios comparing an analysis based on the randomisation strata, the true strata and the updated strata when the interaction effect is of interest and A) errors are equal across strata and error discovery is equal across treatment groups; B) errors are unequal across strata and error discovery is equal across treatment groups; C) errors are equal across strata and error discovery is unequal across treatment groups; and D) errors are unequal across strata and error discovery is unequal across treatment groups. Solid symbols are original simulation results and open symbols are results after changing the sample size from 1000 to 200, or changing the covariate prevalence from 0.5 to 0.75 (if only a solid symbol is observed, results coincide).
